# Supplementary material for: Detection of social anxiety using multiple simultaneous biosignals: A pilot study
Source: PLoS One. 2025 Sep 4;20(9):e0330603. doi: 10.1371/journal.pone.0330603 (PMC12410777; doi:10.1371/journal.pone.0330603)
Supplement: S1 File — (PDF) [file pone.0330603.s001.pdf]

Complete Experimental Protocol

The complete experimental protocol was an adapted version of a study by McFarland et al. [1] where respiratory markers during conversation were investigated.

- Baselines, each 2 minutes: These trials were used to familiarize the participant with the new laboratory environment and the experimental task. It was also used to record signals without interaction with another person.
  - Passive Baseline: The participant sat still and focused on the fixation cross on the screen.
  - Watch and Listen Baseline: The participant watched a video. We used the first 2 minutes from Huberman Lab Clip's youtube channel with the title "How to Properly Hydrate & How Much Water to Drink Each Day — Dr. Andrew Huberman". We picked this video because the creator talks directly to the listener, imitating a one-sided conversation.
  - Read Baseline: The participant read a text aloud after familiarizing themselves with it. We have used the text "Kombat Kate" from the Cambridge English Assessment Example.
  - Free Talk Baseline: The participant talked about a topic of choice without any restrictions.
- Dialogue, 5 minutes each: The dialogues took place between an experimenter and the participant, either in-person or virtually. The order of these two conditions alternated for each participant to avoid any temporal effects and always started with the reading task followed by free talk.
  - In-person Dialogue Reading: For this condition, we used ChatGPT to generate a simple dialogue between two people. While the text had sections of varying lengths for each speaker, the overall length was approximately the same for both, aiming to simulate a realistic conversation with balanced talking and listening parts. For this condition the participant read the dialogue off their smart phone while trying to maintain eye contact with the experimenter when listening.
  - In-person Dialogue Free Talk: For this condition, participants were asked to choose a topic they are familiar with, one that is common enough for the experimenter to ask questions and share their own opinions. The goal for the conversation was again to have approximately equal speaking time for both the participant and the experimenter.
  - Virtual Dialogue Reading: For this condition we have used the same text from above but switched the roles in the dialogue. For this task the experimenter left the room and the talk was performed online using Zoom. For this condition the participant read the dialogue off the LCD screen.

- Virtual Dialogue Free Talk: Same as In-person Dialogue Free Talk with a different topic and using Zoom.

- Anxiety, 5 minutes: The final task was an adapted version of the InterneT-based Stress Test for Social Anxiety Disorder (ITSSAD) by Huneke et al. [2]. Participants were told they would have five minutes to prepare for an online social interaction in which they would have to introduce themselves to a group of researchers. Simply informing participants about the upcoming interview has been shown to increase anxiety, so we did not conduct the full protocol for this pilot. After the five minutes had passed, participants were debriefed and informed that the task was designed to induce elevated levels of social anxiety without the actual interview. Then the experiment was finished.

References

[1] David H McFarland. "Respiratory markers of conversational interaction". In: (2001).

[2] Nathan TM Huneke et al. "A novel procedure to investigate social anxiety using videoconferencing software: A proof-of-concept study". In: *Psychiatry Research* 316 (2022), p. 114770.
