# Supplementary material for: Detection of social anxiety using multiple simultaneous biosignals: A pilot study
Source: PLoS One. 2025 Sep 4;20(9):e0330603. doi: 10.1371/journal.pone.0330603 (PMC12410777; doi:10.1371/journal.pone.0330603)
Supplement: Attachment — Submitted filename: Letter to the reviewer Detection of Social Anxiety.pdf [file pone.0330603.s002.pdf]

Letter to the reviewer for the submission: Detection of Social Anxiety Using Multiple Simultaneous Biosignals: A Pilot Study

July 18, 2025

We offer the following notes on the requested revisions with thanks to our reviewer for taking the time to offer their valuable suggestions to help us improve the quality of the paper. Below is a detailed explanation on how we addressed each comment of the reviewers. Bold text are quotations from the Reviewer.

Referee 1

Major Points

1. **In abstract, manuscript states to test whether anticipatory anxiety can be identified through a combination of signals, on line 39 a 'profile' of features together is to be explored. However, no analysis on combinations or profiles is presented, and no conclusions can be drawn to that end. Here, each signal was tested separately, and limited conclusions can be drawn in current design whether that is due a single latent factor such as vigilance, or due to a specific set of latent variables that may or may not make up a phenotype of SAD. Language should be corrected accordingly to available evidence.**

We have changed the abstract to better reflect the analysis that was performed in this study. **Abstract**: "Social Anxiety Disorder (SAD) is a prevalent and debilitating condition characterized by heightened anxiety and avoidance behaviors in social situations. Despite the availability of treatment options, remission rates for SAD remain low, highlighting the need for more effective interventions. To support the development of more effective therapies, a better understanding of the psychophysiological mechanisms underlying SAD is needed."

We also added more information to the discussion. **Line 355-358**: "However, as this was a pilot study assessing feasibility of our approach, we were unable as yet to conduct analyses on combinations or profiles of signals, which is our ultimate aim. Therefore, our results should be interpreted accordingly."

2. **Methods section is missing part on statistical analysis. Steps to statistical analysis should be explained, and how the problem of family wise error rate was dealt with.**

We have created a new section in the methods called "Statistical Analysis" where we describe the analysis performed in this paper. **Line 173-206**: "Participants were divided into "anxious" and "non-anxious" groups using a median split based on the change in self-reported anxiety from the pre- to post-anticipation phase. Individuals with change scores above the median were categorized as anxious, and those below as non-anxious. This method was chosen to create two approximately equal groups for balanced exploratory analysis in this pilot study.

We analyzed each biosignal modality separately. The figures in the results section that display the biosignals follow a consistent format. The left panel shows the time course of the relevant physiological measure from 30 to 300 seconds. Yellow shaded areas indicate time intervals where statistically significant differences between the two groups were identified using a Wilcoxon rank-sum test. Due to the limited sample size in this pilot study, no correction for multiple comparisons was applied to the time series data. These results are intended to indicate where differences may occur and should be interpreted as exploratory, serving to identify potential time windows of interest for future, larger-scale studies. The right panel of each figure presents a boxplot summarizing the average of the analyzed measure across the full time window. It also includes the p-value for the difference between the groups over this interval, calculated using the same Wilcoxon rank-sum test. For the PSD analysis, significant frequency band differences were found for head roll and EEG data. P-values were displayed uncorrected and corrected using the Benjamini-Hochberg procedure."

3. **Given the small sample and heterogenity of data, descriptive and inferential statistics should be included. Statements that one group had higher score than the other need to be supported by mean/median, STD/IQR, t / F value, and p value. If p value corrections seem too conservative, a permutation approach may help, and authors may present a p value associated with a position of the original t/F value in the distribution of permuted t/F values.**

Based on the recommendations of the reviewer we have added **Table 1** in the results where we report mean, median, std, confidence interval, rank sum value, p-value and Cohen's d. Furthermore, we report the results of head roll (**Figure 10**) and EEG PSD (**Figure 11**) now in a corrected and uncorrected version. We also updated the text of both figures.

Line 256-262: "The PSD plot shows uncorrected p-values on the left, corrected p-values in the middle, and significant corrected p-values on the right. Overall, the PSD of head roll motion shows significant differences between the anxious and non-anxious groups in the 5 Hz to 50 Hz range. The Benjamini-Hochberg correction has only a minor effect, with the corrected results closely matching the uncorrected ones."

Line 263-278: Figure 11 presents EEG PSD values in three panels: the left panel shows PSD differences, the middle displays corrected p-values, and the right shows uncorrected p-values. In the left panel, PSDs are averaged over the entire task duration and across frequency bands, with medians computed across participants. This method reduces the impact of individual variability and improves overall readability. The middle panel applies the Benjamini-Hochberg correction and shows no statistically significant differences. The right panel presents uncorrected p-values and is included to highlight potential differences that may warrant further investigation. Notable uncorrected differences appear in the theta, beta, and gamma bands. Theta band differences are primarily located in central and left temporal regions, while beta and gamma differences are observed in frontal, temporal, and parietal areas."

Minor Points

1. **Intro to abstract reflects need for effective therapies, but not for what the study is doing**

We have added more information how this study can support the development of more effective therapies.

Abstract: "To support the development of more effective therapies, a better understanding of the psychophysiological mechanisms underlying SAD is needed."

Line 19-20: "Higher level Biomedical measures may help identify new therapeutic targets and guide the development of more effective interventions."

Line 363-367: "New, more effective therapies are needed for SAD. In this pilot study we showed that anticipatory social anxiety can trigger changes in a range of biomedical signals namely, respiratory rate, heart rate, tonic GSR, head roll, and central EEG theta activity. We suggest that these signals should be included in montages when studying anxiety and exploring potential options for treatment development."

2. **Sample is not described in abstract**

We have added details about the sample into the abstract. **Abstract:** "Using a modified version of the Internet-based Stress Test for Social Anxiety Disorder, we measured physiological responses of 17 healthy volunteers, including heart rate, respiratory rate, electrodermal activity, head movements, and electroencephalography power across various frequencies."

3. **Conclusion is not linked to abstract introduction**

We have added an additional sentence in the conclusion to link our findings back to our stated aim in the abstract and introduction. **Line 363-367:** "New, more effective therapies are needed for SAD. In this pilot study we showed that anticipatory social anxiety can trigger changes in a range of biomedical signals namely, respiratory rate, heart rate, tonic GSR, head roll, and central EEG theta activity. We suggest that these signals should be included in montages when studying anxiety and exploring potential options for treatment development."

4. **Line 8 unclear how cost is related to SAD**

We have added more information how the cost highlights the impact of social anxiety disorder. **Line 8-12:** "To our knowledge, this was the most recent formal estimate, but it is likely that current costs are even higher. Quantifying these economic burdens highlights the significant impact of anxiety disorders on public health systems and underscores the urgency of improving prevention and treatment strategies."

5. **Line 23 - personal believes should be based on preliminary evidence or case reports**

We rephrased the comment. **Line 28-30:** "Given these findings, physiological reactivity and interoceptive awareness might interact to maintain anxiety."

6. **Line 42, accronym EDA is not explained**

Electrodermal activity is spelled out in **line 39**.

7. **Line 72, experimental procedure, high-pass analog filter (on recording device) should be included, especially when interpreting very slow oscillations**

We have added this information to the experimental procedure section. **Line 89-90:** "All of the signals recorded by the actiChamp plus system underwent on-device analog high-pass filtering at 0.016 Hz."

8. **From the description of experimental task, in the supplementary material, or original paper by Huneke I cannot figure when was the GAD administered. Was it before the task was introduced? and Before it was revealed that experiment is not going to include interview, or after that? I would appreciate clarification on this to understand the results.**

We have added that information to the experimental task section. **Line 110-111:** "The first GAD7 was introduced before introducing participants to the anxiety task."

9. **Line 109 - Could you elaborate on how did you split the group? I can clearly see that some people in anxious groups have lower values on GAD than in non-anxious in Fig2. This should be in the part Statistical analysis. This part may also include what significance test was used and why, and how was treated the multiple comparisons problem**

We have added a better description of the participant split in the methods and provided a clearer plot in the results. **Line 174-184:** "Participants were divided into "anxious" and "non-anxious" groups using a median split based on the change in self-reported anxiety from the pre- to post-anticipation phase. Individuals with change scores above the median were categorized as anxious, and those below as non-anxious. This method was chosen to create two approximately equal groups for balanced exploratory analysis in this pilot study."

10. **line 160 - could add 'Head roll' for clarity**

We have changed this in **line 134** and **167** as well as in the caption in **figure 10** and the figure itself.

11. **Line 162 - signal filtered 0.5 - 50Hz, but downsampled to 100Hz, while this satisfies nyquist frequency, higher frequencies should be interpreted cautiously, as caution commands that frequencies higher than 1/3 or 1/4th of sampling frequency should not be analysed.**

After further research and contacting additional experts we are not aware of such interpretation. In the discussion (**line 312-316**) we already highlight that we assume that activity in the beta and gamma band (>20 Hz) is likely not related to brain but muscle activity. Would it be possible to provide literature that describes this phenomenon if additional clarification is necessary?

12. **Lines 169-170. Claims higher or lower should be accompanied by descriptive statistics and inferential statistics**

We removed that plot and description and replaced it with a clearer version. **Figure 2 + Line 208-211:** "The boxplots in Figure 2 show the differences in GAD-7 scores, averaged across all questions, for anxious and non-anxious participants from before to after the anxiety condition. Participants whose score differences were below the median were classified as non-anxious, while those above the median were classified as anxious."

13. **Fig 2 description may for clarity include how groups were split to be stand-alone understandable**

We replaced figure 2 with a clearer figure and replaced the caption. **Figure 2 caption:** "The boxplots in Figure 2 show the differences in GAD-7 scores, averaged across all questions, for anxious and non-anxious participants from after to before the anxiety condition. Participants whose score differences were below the median were classified as non-anxious, while those above the median were classified as anxious."

14. **Lines 175 to 179 belong to methods**

We have remove this paragraph and added the information to the statistical analysis section in the methods.

15. **Fig 10. Head rolls need more explanation. Head motion is traditionally measured as oscillation in eg parkinsons, or head boobing and sway is measured below 5Hz. Oscillations of head are not usually present, and do not appear meaningful. This result is intriguing, but without further context, and without correction for multiple comparisons, may be spurious. Perhaps a histogram or boxplot of values averaged over significant frequencies would provide further credibility?**

We added additional information for the head roll analysis. Figure 10 now displays correct and uncorrected p-values and we changed the methods and result texts. **Line 204-206:** "For the PSD analysis, significant frequency band differences were found for head roll and EEG data. P-values were displayed uncorrected and corrected using the Benjamini-Hochberg procedure." **Line 256-262:** "The PSD plot shows uncorrected p-values on the left, corrected p-values in the middle, and significant corrected p-values on the right. Overall, the PSD of head roll motion shows significant differences between the anxious and non-anxious groups in the 5 Hz to 50 Hz range. The Benjamini-Hochberg correction has only a minor effect, with the corrected results closely matching the uncorrected ones."

16. **Line 247 - 'marginally significant' is not meaningful. Size of an effect is not measured by a p value.**

We removed parts of these sentences and rephrased the rest.

Line 293-296: "A study by Pittig et al. [1], for example, showed that the reduction in heart rate variability (HRV) in individuals with social anxiety disorder (SAD) was weaker compared to other anxiety disorders."

17. **Line 277 - If it was not significant, there was no difference. Sometimes, in pilot data, we can speak about 'trends'. However, fig 5. right appears to show homogenous groups and one extreme value.**

We have changed the line in the discussion. **Line 324-326:** "We did not find a significant difference between groups in pupil size."

References

[1] A. Pittig, J. J. Arch, C. W. Lam, and M. G. Craske, "Heart rate and heart rate variability in panic, social anxiety, obsessive–compulsive, and generalized anxiety disorders at baseline and in response to relaxation and hyperventilation," *International journal of psychophysiology*, vol. 87, no. 1, pp. 19–27, 2013.
